# An examination of nucleotypic effects in diploid and polyploid cotton

**DOI:** 10.1093/aobpla/plw082

**Published:** 2016-12-24

**Authors:** S.J. Snodgrass, J. Jareczek, J. F. Wendel

**Affiliations:** 1 Department of Biology, Grinnell College, Grinnell, IA, USA; 2 Department of Ecology, Evolution, and Organismal Biology, Iowa State University, Ames, IA, USA

**Keywords:** Cell size, genome size, Gossypium, guard cell, polyploidy, stomata

## Abstract

Nucleotypic effects are phenotypic changes related to the total nuclear DNA amount per cell. These effects are commonly observed among and within genera for certain cell types, and the generality of the positive correlation between genome size and cell size has been well established. However, there are few studies of nucleotypic effects which incorporate into the analysis both ploidy level and genome size (given as Mbp determined by 2C values). To test the hypothesis that cell size scales with genome size and ploidy, we measured the guard cell length, epidermal pavement cell surface area, and pollen grain diameter using individuals of multiple species and accessions of the cotton genus (*Gossypium*), in which different species exhibit three-fold variation in genome size. We measured cell sizes using calibrated microscopic image analysis. Significant relationships were found between genome size and cell size, with stronger correlations between guard cell length and genome size than with epidermal pavement cell surface area. We also found a relationship between pollen grain diameter and genome size. These results indicate that nucleotypic effects occur within *Gossypium*, scale with ploidy level, and are stronger in less variable cell types.

## Teaser

This paper outlines the relationship between genome size and cell size in diploid and polyploid cotton plants, reinforcing previous observations that larger genomes correlate with larger cells in plants. We also note that domesticated cotton plants have smaller cells than their wild counterparts and that nucleotypic effects seem stronger in more specialized cell types.

## Introduction

Nucleotypic effects—changes in phenotype related to the total amount of nuclear DNA within and among taxa—are well-documented in plant biology, with reports tracing to as early as the 1930s (Davis and Heywood 1963). Correlative studies across taxa have found that nuclear DNA amount may be positively correlated with cell or seed size (Anderson *et al*. 1985; Beaulieu *et al*. 2008; Hodgson *et al*. 2010; Knight 2005; Knight and Beaulieu 2008; Otto 2007; Rees *et al*. 1966; Vesely *et al*. 2012), and negatively correlated with stomatal density (Beaulieu *et al*. 2008; Knight and Beaulieu 2008). Studies within single genera or species have found more variable results, including differences between various cell types. In *Arabidopsis*, a positive correlation between ploidy and size of guard cells, epidermal pavement cells, and trichomes has been reported, with epidermal pavement cells being more variable than other cell types (Melaragno *et al*. 1993). A different study of *Arabidopsis* focusing on leaf mesophyll, petal epidermis, and pollen grains, however, did not find a correlation between ploidy and cell size (Tsukaya 2013). Despite this, the positive correlation between genome size and cell size is often observed (Cavallini *et al*. 1993; Coate *et al*. 2012; Kenton *et al*. 1986; McCarthy *et al*. 2015; Mishra 1997; Mowforth and Grime 1989; Wong and Murray 2012).

Edwards and Endrizzi (1975) surveyed 17 species of *Gossypium* and reported a positive correlation between DNA content and cell volume for a single cell type, i.e., those from the meristematic (distal 0.5 mm) region of root tips. It is unknown whether this pattern holds for other cell types or for cells having different ploidy levels. To address the questions of how nucleotypic effects scale with polyploidy and nucleotypic differences between cell types, we explored the relationships among genome size and several cellular features in *Gossypium* diploids and allopolyploids, specifically guard cell size, epidermal pavement cell (EPC) surface area, and pollen grain diameter. We used *Gossypium* as a model because of the three-fold genome size variation that exists among the diploid species, and because of its well-established evolutionary framework for the allopolyploid formation (Wendel and Grover 2015). Notably, A- and D-genome species differ two-fold in genome size, with near-additivity in the allopolyploids. We hypothesized that species with larger genomes will have larger cell sizes (AD >A >D) and that accessions from the same genome group will have similar cell sizes. Because mature cell shapes and volumes for some cell types may be developmentally more plastic than other cell types, contingent upon, for example, environmental conditions during growth, we also hypothesized that nucleotypic effects will be stronger for cell types that are more canalized or homogeneous in their size (guard cells, pollen grains) than for more variable cell types (here, EPCs), as in Melaragno *et al*. (1993).

## Methods

### Cell type choices

We chose to measure guard cells and EPCs because they are readily accessible and uniformly present among *Gossypium* species and accessions, unlike other cell types, such as non-glandular trichromes. Guard cells and EPCs also span a range of intraspecific variability in size and shape. Pollen grains were included because they are uniform in shape and are readily collected during flowering times. Subsidiary cells were not sampled due to their variability in number (Wullschleger and Oosterhuis 1989) and patterning (Bondada and Oosterhuis 2000; Gangadhara *et al*. 1977).

### Plant material

The ‘A genome’ diploid ancestor of allopolyploid (‘AD genome’) cottons, from the Old World, and the ‘D genome’ diploid ancestor, from the New World, hybridized 1-2 million years ago to form a new allopolyploid clade in the New World (Wendel and Grover 2015). Two different allopolyploid and two different diploid species were independently domesticated for their abundant seed epidermal trichomes (cotton ‘fiber’), which can be harvested and spun into fabrics. All plant materials studied came from the collection of *Gossypium* maintained by the Wendel lab in the Pohl Conservatory at Iowa State University. All plants were grown under identical greenhouse conditions, in rooms that received natural light and with temperatures held at 80^°^ F during the day, and 72^°^F at night. For leaf tissues, the following accessions were sampled: MAXXA, Yucatanese (abbr. YUC), A1-73, WAGAD, A2-101, and D5. MAXXA and YUC are allopolyploid accessions of *G. hirsutum,* the former domesticated and the latter wild. A1-73 and WAGAD, respectively, are wild and domesticated forms of the diploid species *G. herbaceum.* A2-101 is a domesticated diploid accession of the species *G. arboreum*. D5 is a wild diploid accession of the species *G. raimondii*. Thus, three genome groups were included in the study, the A (A1-73, WAGAD, and A2-101), AD (MAXXA, Yucatanese), and D (D5) (Table 1).

**Table 1.** Accessions sampled for leaf tissue and pollen grains. Genome size information is from Hendrix and Stewart (2005) rounded to the nearest 100 mbp.

Genome Group	Species	Accession	Ploidy Level	Domestication Status	Genome Size (Mbp)	Material Collected
AD1	*G. hirsutum*	MAXXA	tetraploid	domesticated	2400	leaf, pollen
AD1	*G. hirsutum*	Yucatanese (abbr. YUC)	tetraploid	wild	2400	leaf
AD1	*G. hirsutum*	TX2094	tetraploid	wild	2400	pollen
A1	*G. herbaceum*	WAGAD	diploid	domesticated	1700	leaf
A1	*G. herbaceum*	A1-73	diploid	wild	1700	leaf
A1	*G. herbaceum*	A1-79	diploid	wild	1700	pollen
A2	*G. arboreum*	A2-101	diploid	wild	1700	leaf, pollen
D5	*G. raimondii*	D5	diploid	wild	880	leaf, pollen

Pollen grain samples were taken from flowering individuals of the same accessions with a few exceptions. Individuals of WAGAD were not in flower and no individuals of similar accessions were flowering; thus, pollen grains from individuals of domesticated *G. herbaceum* were not included. Similarly, A1-73 was not in flower, but we did sample individuals of a genetically similar accession, A1-79.

### Leaf feature measurements

We considered the following possible sources of variation in our design: sampling surface, leaf, and individual. While leaves in *Gossypium* are amphistomatal, the abaxial surface exhibits greater stomatal density than the adaxial surface (Wise *et al*. 2000; Wullschleger and Oosterhuis 1989), as is commonly observed in plants. It was also found that guard cells of the adaxial surface were generally larger than those of the abaxial surface in *Gossypium* (Bondada and Oosterhuis 2000) and in other genera (Zarinkamar 2007). However, Wise *et al*. (2000) found no difference between the guard cell measurements of the abaxial and adaxial surfaces in *G. hirsutum*. To account for variation among leaves within individuals, we selected two leaves from each plant. Similarly, to account for variation among individuals within accessions, we sampled three different individuals. Thus, for each accession studied (Table 1), two fully expanded, mature leaves from each of three individuals were taken from similar positions within the canopy.

**Figure 1a:**
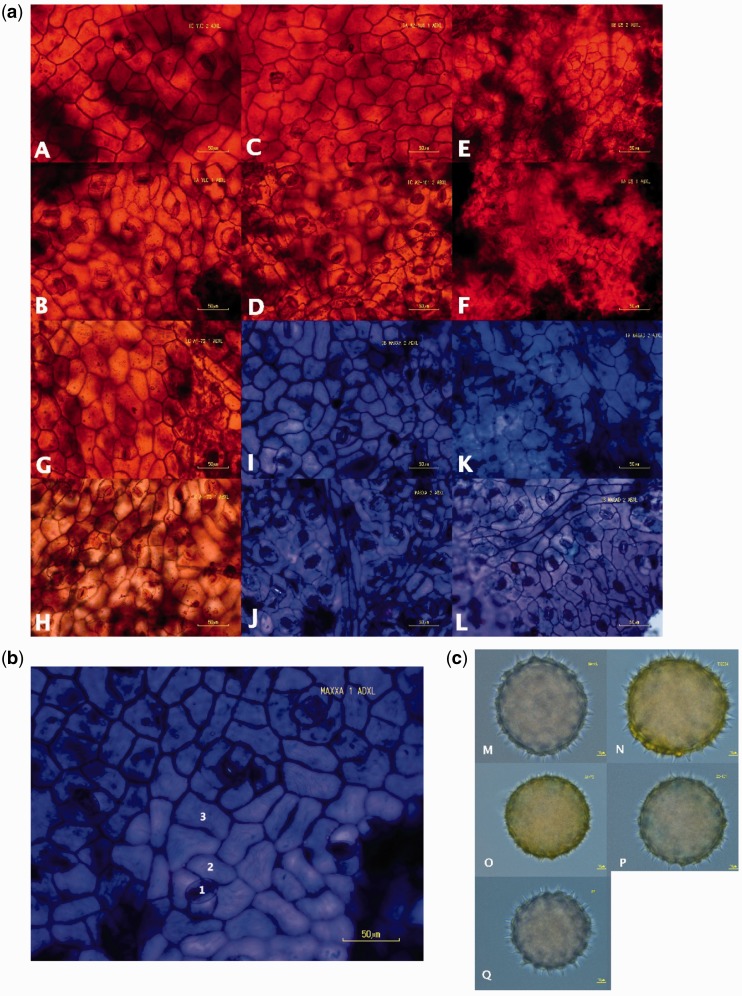
(A) Images of leaf epidermal peels (scale bars = 50 μm). Red and blue images are peels stained with Safronin and Toluidine, respectively. (A) *G. hirsutum* race *yucatanese* adaxial surface (B) *G. hirsutum* race *yucatanese* abaxial surface (C) *G. arboreum* A2-101 adaxial surface (D) *G. arboreum* A2-101 abaxial surface (E) *G. raimondii* adaxial surface (F) *G. raimondii* abaxial surface (G) *G. herbaceum* subsp*. africanum* A1-73 adaxial surface (H) *G. herbaceum* subsp*. africanum* A1-73 abaxial surface (I) *G. hirsutum* cv. MAXXA adaxial surface (J) *G. hirsutum* cv. MAXXA abaxial surface (K) *G. herbaceum* cv. Wagad adaxial surface (L) *G. herbaceum* cv. Wagad abaxial surface. **1b:** Image of *G. hirsutum* MAXXA adaxial leaf epidermis. Numbers correspond to cell types. (1) stomate (2) subsidiary cell (3) epidermal pavement cell. **1c:** Pollen grains of five accessions (scale bars = 10 μm). (M) *G. hirsutum* cv. MAXXA (N) *G. hirsutum* cv. TX2094 (O) *G. herbaceum* subsp. *africanum* A1-79 (P) *G. arboreum* A2-101 (Q) *G. raimondii*.

Epidermal peels were taken from both the abaxial and adaxial surfaces. The peels were created using the scraping procedure of Cutler *et al*. (2008). The peels were stained with either Toluidine Blue or Safronin, depending on availability, and mounted for temporary use on glass slides (both stains are equally efficacious for the purposes of this study). Images of peels were taken using a phase contrast microscope (Nikon Eclipse SSi, Nikon Metrology, Brighton, Michigan, USA) and imaging system (Nikon Digital Sight, Nikon Metrology), using tissue from the more basal quadrants of the leaves. All images were taken at 200x magnification (Fig. 1A).

Image features were quantified using ImageJ software (http://imagej.nih.gov/ij/index.html). Guard cell length was measured along the long axis of the stomate and epidermal cell surface area was measured using the outline of the cell. All stomata that were clearly and fully within the borders of each image were measured. Due to the challenge of clearly differentiating subsidiary cells from EPC, cells that were not touching stomata, that were fully in view, and that did not touch a previously measured cell were considered EPCs, and were measured (Fig. 1B). The count range for guard cells was 498 cells to 952 cells. The count range for EPCs was 614 cells to 1107 cells. Leaves from some accessions produced better epidermal peels than others, resulting in variation in cell counts (Fig. 1).

### Pollen measurements

Pollen was collected from similar or identical accessions as for the leaf material (Table 1, Fig. 1C). Pollen grains were stained with lactophenol-aniline blue and mounted on slides. Twenty pollen grains were photographed from an individual of each accession at 400x magnification on a phase contrast microscope and imaging system. The pollen grain diameter was measured using ImageJ software. The diameter was considered from the tip of a spine to the tip of an opposing spine. If a measurement couldn’t be made vertically because part of the grain was not imaged, horizontal diameter was measured.

### Correlation between guard cell surface area and guard cell length

Twenty abaxial guard cells per leaf were sampled for length and surface area. Our definition of surface area was the area encompassed by both guard cells around a single stomate. Length was measured as described above. Measures of the same guard cells were paired for testing correlation of guard cell length and stomatal surface area. The goal was to account for stomatal opening as a source of variation.

### Statistical methods

Because the experiment nested surface within leaf within individual within accession, surface is the lowest independent level of the model with individual cells acting as repeated measurements within each nested category. The average of each category was used for analysis of variance of the 72 combinations of 6 accessions, 3 individuals, 2 surfaces, and 2 leaves.

We used the genome sizes published in Hendrix and Stewarts (2005) to group individual accessions for orthogonal contrasts to determine differences between accessions differing in genome size. The General Linear Model function of the Minitab 17 software was used for statistical analyses. *R*^2^_adjusted_ values are reported for each of these models; this takes the number of predictor variables into account when calculating the *R*^2^. Contrasts were performed using the procedure for orthogonal contrasts in Oehlert (2000). *P*-values less than 0.05 are considered significant.

## Results

### Guard cell length

**Figure 2:**
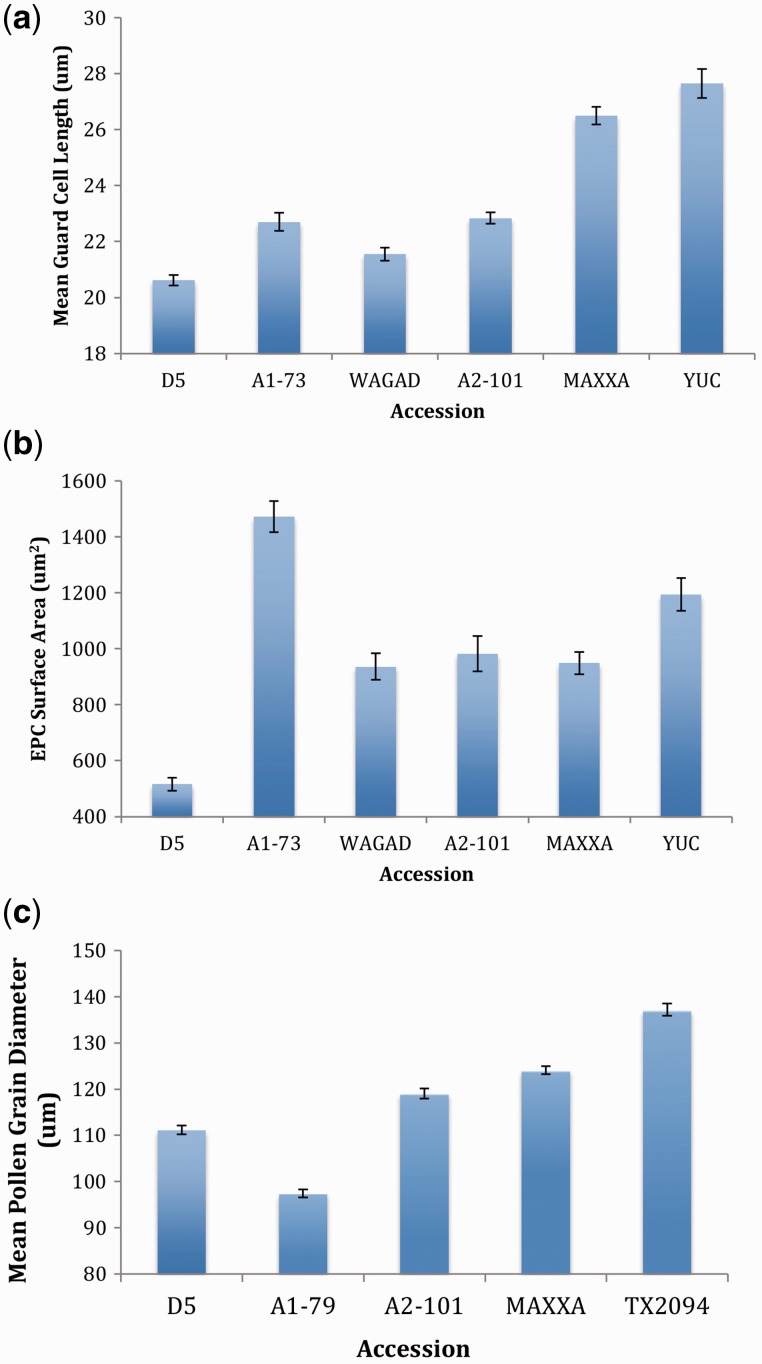
All bars represent one standard error. **(a)** Mean guard cell length by accession. **(b)** Mean epidermal pavement cell surface area by accession. **(c)** Mean pollen grain diameter by accession.

The model for guard cell length found all nested factors to be significant (accession *F =* 23.21, *P <* 0.001; individual *F =* 3.37, *P =* 0.010; leaf *F =* 4.32, *P <* 0.001; Table 2) and accounted for 96.4 % of the variation (*R*^2^_adjusted_). The orthogonal contrasts found a significant difference between plants from the AD genome group (MAXXA and Yucatanese) and those from the diploid A and D genomes (A1-73, Wagad, A2-101, D5) (*P <* 0.001), with the AD plants having longer guard cells than plants from the A and D genomes (AD > A, D). Additionally, the contrasts indicated a significant difference between the A and D genome groups (*P =* 0.0486), with A having a longer guard cells than D (A > D, Fig. 2A). There was no significant difference between Yucatanese and MAXXA (*P =* 0.2187), nor between A1-73 and Wagad (*P =* 0.2196).

**Table 2.** ANOVA Models for each cell type using Minitab17. Accession is fixed, all other variables are random.

Cell type	Guard Cell Length				
Source	Type III sum of squares	df	Mean square	*F*	*P*
Accession	470.46	5	94.0914	23.21	0.000
Individual (Accession)	48.65	12	4.0542	3.37	0.010
Leaf (Accession, Individual)	21.68	18	1.2043	4.32	0.000
Error	10.05	36	0.2791		
Total	550.83	71			
**Cell type**	**Epidermal Pavement Cell Surface Area**				
**Source**	**Type III sum of squares**	**df**	**Mean square**	***F***	***P***
Accession	6021888	5	1204378	13.87	0.000
Individual (Accession)	1041979	12	86832	2.75	0.026
Leaf (Accession, Individual)	568042	18	31558	3.15	0.002
Error	360452	36	10013		
Total	7992360	71			
**Cell type**	**Pollen Grain Diameter**				
**Source**	**Type III sum of squares**	**Df**	**Mean square**	***F***	***P***
Accession	17542	4	4385.59	204.47	0.000
Error	2038	95	21.45		
Total	19580	99			

### Epidermal pavement cell surface area

The model for EPC surface area found all nested factors to be significant (accession *F =* 13.87, *P <* 0.001; individual *F =* 2.75, *P =* 0.026; leaf *F =* 3.15, *P =* 0.002; Table 2) and accounted for 91.1 % of the variation (*R*^2^_adjusted_). Orthogonal contrasts were not significantly different between the polyploids and diploids (*P =* 0.2564), but were for the A versus D genome groups (*P =* 0.0015) (A > D, Fig. 2B). No significant difference was detected between the two *G. hirsutum* accessions, Yucatanese and MAXXA (*P =* 0.970) but there was a difference between the two *G. herbaceum* accessions A1-73 and Wagad (A1-73 > Wagad) (*P =* 0.0067).

### Pollen grain diameter

The model found accession to be a significant factor (f = 1204.47, *P <* 0.001; Table 2) and accounted for most of the variation (*R*^2^_adjusted _=_ _89.2 %). Orthogonal contrasts indicate a significant difference between the polyploids and diploids (*P <* 0.001) but not between the two diploid genome groups (*P =* 0.1440) (AD > A, D; Fig. 2C). The two *G. hirsutum* accessions had significantly different pollen grain diameters (Tx2094 > MAXXA) (*P <* 0.001).

## Discussion

### Correlation between cell sizes and genome size

We predicted that accessions with larger genome sizes would also have larger cells, following the pattern of AD > A > D, with accessions of the same genome group showing no differences. This pattern held for guard cell length and pollen grain diameter, but not for EPC surface area. These results parallel those of Katagiri *et al*. (2016), indicating that cell type is a factor in the relationship between nuclear DNA amount and cell size.

Guard cell length and guard cell surface area were highly correlated (R-sq = 0.88), indicating that stomate aperture influences the guard cell length, as previously reported (Meckel *et al*. 2007). We observed a small effect, such that for every 1 μm^2^ increase in surface area, there was a 0.026 μm increase in length.

The EPC surface area did not clearly demonstrate nucleotypic effects. Cells from the A and D genome group accessions were different from one another, but were not different from those of the AD tetraploids. The former of these two results likely reflects the inclusion of the *G. herbaceum* accession, A1-73; these individuals had larger EPC surface areas than individuals of the tetraploid *G. hirsutum* accessions Maxxa or Yucatanese (Fig. 2). This, in addition to similar surface areas of Wagad, A2-101, and MAXXA, likely accounts for the absence of significant difference between the polyploids and diploids in the orthogonal contrasts. The difference between A1-73 and the other A genome accessions was unexpected. However, interspecific and intraspecific genetic variation within the A genome group is extensive (Kebede *et al*. 2007; Wendel *et al*. 1989). Moreover, accession A1-73 is the only truly wild A-genome sample studied, and it is both geographically and genetically distant from the others. Additional sampling within this genome group will be necessary to assess levels and patterns of variation in the features studied here.

Pollen grain diameters were consistent with our nucleotypic hypotheses, with some deviation. Pollen from polyploid accessions did have larger diameters than did pollen from diploid accessions, and pollen diameter was significantly different between wild and domesticated polyploid accessions. This latter difference may be a domestication effect. However, pollen grains of diploid accessions did not follow nucleotypic trends, with the smaller D5 genome having larger pollen than that of the wild A genome accession, A1-79 (Fig. 2C). This indicates that while nucleotypic effects may influence the pollen grain diameter, other factors such as domestication (possibly illustrated by the larger A2-101 pollen diameter) are also important. It has also been shown by Knight *et al*. (2010) that pollen grains do not always display nucleotypic effects.

### Patterns of cell size between domesticated and wild accessions

While there were no differences in guard cell length between domesticated and wild accessions of the same species, there were differences in the EPC surface area and in pollen grain diameter. With regards to the former, A1-73 and Wagad were significantly different, with A1-73 having larger cells than those of the domesticated accession Wagad (*P =* 0.0067). With respect to pollen grain diameter, TX2094 individuals had larger pollen grains than did MAXXA individuals (*P <* 0.001). This indicates that for at least a couple of cell types, domesticated accessions have smaller cells compared to wild counterparts of the same species. Previous studies of domestication effects on cell size have mostly focused on fruit and seed enlargement, often reporting that the number of cells as well as cell volume increase as a result of domestication (Colunga-GarciaMarin *et al*. 1996; Guo and Simmons 2011; Malladi and Hirst 2010; Schwanitz 1966). Evans (1976) suggests an increase in leaf size relates to an increasing cell size, whereas Reinhardt and Kuhlemeier (2002) suggest both cell division and cell enlargement are key to the final leaf size. Also, there may be interdependence of cell division and enlargement (del Pozo and Ramirez-Parra 2015; Reinhardt and Kuhlemeier 2002), with both of these features under complex regulatory control (Guo and Simmons 2011). Distribution and size changes in stomata of domesticated plants have been observed (Milla *et al*. 2013), perhaps reflecting changing water availability between cultivated fields and wild settings. We did not, however, find a difference in guard cell length between the two domesticated-wild pairs studied here. With the caveat that only a single accession was sampled for each class (wild, domesticated) for each of these two domesticated cotton species, our results are only preliminary, and raise the possibility that domestication has led to smaller epidermal pavement cells and pollen grains, without changes in guard cell length.

### Possible mechanisms for nucleotypic effects

It is hypothesized that nucleotypic effects in plants occur because cells with larger genomes require larger nuclei, with cascading effects related to overall cell size (Price 1988). Nucleotypic effects may also have complex regulatory underpinnings (del Pozo and Ramirez-Parra 2015), as evidenced by the specificity of cell identity as an important determinant for genome size-cell size relationships in *Arabidopsis thaliana* (Katagiri *et al*. 2016). At present, much remains to be learned regarding the multitude of factors that determine cell sizes in plants, and the cases in which a direct relationship to genome size is important.

## Conclusions

Nucleotypic effects appear to be acting upon guard cell length, EPC surface area, and pollen grain diameter in *Gossypium*. These effects are strongest on guard cell length and pollen grain diameters. In addition to a general increase in cell sizes with an increase in genome size, there is evidence of differences between cells from domesticated and wild accessions of the same species. The individuals of domesticated accessions have smaller cells than their wild counterparts, possibly indicating some effect of the domestication process and water availability. However, more domesticated and wild pairs would have to be studied to confirm this relationship.
